# Early assessment of lung function in coronavirus patients using invariant markers from chest X-rays images

**DOI:** 10.1038/s41598-021-91305-0

**Published:** 2021-06-08

**Authors:** Mohamed Elsharkawy, Ahmed Sharafeldeen, Fatma Taher, Ahmed Shalaby, Ahmed Soliman, Ali Mahmoud, Mohammed Ghazal, Ashraf Khalil, Norah Saleh Alghamdi, Ahmed Abdel Khalek Abdel Razek, Eman Alnaghy, Moumen T. El-Melegy, Harpal Singh Sandhu, Guruprasad A. Giridharan, Ayman El-Baz

**Affiliations:** 1 BioImaging Laboratory, Department of Bioengineering, University of Louisville, Louisville, KY USA; 2 College of Technological Innovation, Zayed University, Dubai, UAE; 3 Department of Electrical and Computer Engineering, Abu Dhabi University, Abu Dhabi, UAE; 4 College of Computer and Information Science, Princess Nourah bint Abdulrahman University, Riyadh, Saudi Arabia; 5 Department of Diagnostic Radiology, Faculty of Medicine, Mansoura University, Mansoura, Egypt; 6 Faculty of Engineering, Assiut University, Assiut, Egypt; 7 Department of Ophthalmology and Visual Sciences, University of Louisville, Louisville, KY USA

**Keywords:** Biomarkers, Diseases, Biomedical engineering

## Abstract

The primary goal of this manuscript is to develop a computer assisted diagnostic (CAD) system to assess pulmonary function and risk of mortality in patients with coronavirus disease 2019 (COVID-19). The CAD system processes chest X-ray data and provides accurate, objective imaging markers to assist in the determination of patients with a higher risk of death and thus are more likely to require mechanical ventilation and/or more intensive clinical care.To obtain an accurate stochastic model that has the ability to detect the severity of lung infection, we develop a second-order Markov-Gibbs random field (MGRF) invariant under rigid transformation (translation or rotation of the image) as well as scale (i.e., pixel size). The parameters of the MGRF model are learned automatically, given a training set of X-ray images with affected lung regions labeled. An X-ray input to the system undergoes pre-processing to correct for non-uniformity of illumination and to delimit the boundary of the lung, using either a fully-automated segmentation routine or manual delineation provided by the radiologist, prior to the diagnosis. The steps of the proposed methodology are: (i) estimate the Gibbs energy at several different radii to describe the inhomogeneity in lung infection; (ii) compute the cumulative distribution function (CDF) as a new representation to describe the local inhomogeneity in the infected region of lung; and (iii) input the CDFs to a new neural network-based fusion system to determine whether the severity of lung infection is low or high. This approach is tested on 200 clinical X-rays from 200 COVID-19 positive patients, 100 of whom died and 100 who recovered using multiple training/testing processes including leave-one-subject-out (LOSO), tenfold, fourfold, and twofold cross-validation tests. The Gibbs energy for lung pathology was estimated at three concentric rings of increasing radii. The accuracy and Dice similarity coefficient (DSC) of the system steadily improved as the radius increased. The overall CAD system combined the estimated Gibbs energy information from all radii and achieved a sensitivity, specificity, accuracy, and DSC of 100%, 97% ± 3%, 98% ± 2%, and 98% ± 2%, respectively, by twofold cross validation. Alternative classification algorithms, including support vector machine, random forest, naive Bayes classifier, K-nearest neighbors, and decision trees all produced inferior results compared to the proposed neural network used in this CAD system. The experiments demonstrate the feasibility of the proposed system as a novel tool to objectively assess disease severity and predict mortality in COVID-19 patients. The proposed tool can assist physicians to determine which patients might require more intensive clinical care, such a mechanical respiratory support.

## Introduction

Coronavirus disease 2019 (COVID-19) was initially detected in Wuhan, China and is caused by a novel RNA virus belonging to the Coronaviridae family. It is believed to have been transmitted to humans from bats via an intermediate mammalian host before achieving human to human transmission. Such zoonotic origin is consistent with similar coronavirus outbreaks^1–4^. Coronaviridae is a family of nonsegmented, enveloped, positive-sense, single-stranded ribonucleic acid viruses. Six species of coronavirus had previously been identified as pathogenic in humans: four of these cause mild respiratory illnesses, whereas the other two species, severe acute respiratory syndrome coronavirus (SARS-CoV) and Middle East respiratory syndrome coronavirus (MERS-CoV), have led to epidemics with significant rates of mortality^5^.

The clinical diagnosis of COVID-19 depends on different symptoms including fever in 98% of cases, dry cough (75%), fatigue (45%), muscle aches (45%), difficulty breathing (55%), and acute respiratory distress syndrome (ARDS) (20%). Severe cases may progress to multiorgan dysfunction and even death (2.5%)^4,6–8^. The disease may be classified as (i) mild type: moderate clinical symptoms with normal chest X-ray; (ii) typical type: fever, respiratory, and other clinical findings indicating signs of pneumonia; (iii) severe type: respiratory distress signs (respiratory rate $\ge 30$ breaths per minute and/or blood oxygen saturation of less than 93%; (iv) critical type: dysfunction of respiration necessitating mechanical ventilation, shock, and organ damage requiring monitoring and treatment from the intensive care unit (ICU)^9^.

Due to the wide variations in clinical presentation and progression rate for COVID-19, laboratory confirmation of SARS-CoV-2 infection is essential to initiate appropriate early treatment and to prevent further spread of the disease^10–15^. The current reference standard for this purpose is real-time reverse transcription polymerase chain reaction (PCR) of viral RNA. The PCR test, according to current guidelines, is run on samples from nasopharyngeal and/or throat swabs. While PCR is the gold standard in diagnosing patients with COVID-19 infection, the sensitivity of a single PCR is suboptimal and depends on the timing of the test, sampling sites and sampling techniques^7,8,10–12^.

Chest radiography is helpful for first-line evaluation of patients with a high pre-test probability of overt COVID-19 pneumonia, clinical follow up, and for the evaluation of potential complications. Chest radiography can detect areas of ground glass density, also observed on chest computed tomography (CT), which may often have a correlation with the severity of the disease, and may be intermixed with reticular pattern^12–17^.

Based on the recent clinical research, COVID-19 radiological forms are variable in severity using plain radiography or CT, ranging from a normal chest (albeit rarely), to patchy involvement of one or both lungs in mild or moderate cases, to diffuse infiltration (white lung) in severe cases. This is an important issue as mild or moderate cases can be managed by medical treatment or non-invasive ventilation, while severe cases with bilateral lung infection urgently need mechanical ventilation to support respiration as patients develop ARDS. Given the paucity of mechanical ventilation units, patient selection for ventilation plays a crucial role in saving lives. We propose a methodology that utilizes the current state of machine learning and artificial intelligence (AI) to assist physicians by providing an objective metric that can differentiate severe cases from mild/moderate cases and potentially even predict mortality.

There are few preliminary studies and case reports discussing the role of AI on plain radiography and CT for early diagnosis of patients with COVID-19. AI can be used in conjunction with radiologists to improve the results of detection of COVID-19. AI can be a powerful aid in delineating and quantifying lesions in X-ray images and in tracking longitudinal changes between exams, which is crucial for precision medicine. In essence, AI is another means of analyzing data that clinicians can draw on to inform their judgment in issues of triage, diagnosis (in combination with PCR tests and epidemiological risk), prognosis, and selection between therapeutic alternatives in patients exhibiting COVID-19 symptoms. Plain radiography involves a low radiation dose compared to CT and is better suited for routine monitoring and follow up compared to a CT scan. AI may be capable of detecting subtle changes visible on either chest X-ray or CT in the lung, and can improve efficiency by decreasing the amount of time to return test results. This is necessary for screening the general population during the current COVID-19 pandemic and in the epicenters of any future outbreaks. Computer assisted detection alleviates the burden on radiologists and clinicians and facilitates rapid triage. Also, AI can be used for the differentiation of previous lung injury unrelated to COVID-19 from advanced lung dysfunction due to COVID-19, and assist in patient selection for ventilation^18–25^.

CAD systems for assessing lung function in COVID-19 are limited in the literature. Sun et al.^26^ developed an approach using deep transfer learning to detect signs of COVID-19-related pneumonia in chest X-ray images. Hassanien et al.^27^ developed an automatic segmentation method for lung areas affected by COVID-19 that employed an Otsu-derived algorithm for multi-level thresholding of X-ray images and a support vector machine for the prediction task. Apostolopoulos et al.^28^ studied the efficacy of deep learning convolutional neural networks (CNN) for deriving characteristic COVID-19 biomarkers from chest X-rays. Wang et al.^29^ presented a novel CNN, named COVID-Net, tailored to the detection of COVID-19-related changes in chest X-rays. The deep learning network of Hammoudi et al.^30^, on the other hand, was designed to automatically detect if a chest X-ray image indicates healthy lungs or evidence of pneumonia (bacterial or viral). Combined with prior information regarding the likelihood the patient has been exposed to the virus, an automatic diagnosis of viral pneumonia has a high true positive rate for detection of COVID-19.

Currently, the primary challenge is to apply different AI-based approaches to determine the severity of chest infection in COVID-19 patients given that X-ray images vary enormously in image quality due to the wide range of X-ray machines in use across the world. To overcome this challenge, we develop a new CAD system that operates on extracted X-ray image markers that are invariant under rotation, scaling, and translation.

## Materials and methods

### Patient data

The proposed approach is tested and validated on data from a publicly available archive of COVID-19 positive cases^31^, data from COVID-19 open research dataset challenge (CORD-19)^32^, and data from the University of Louisville, USA and Mansoura University, Egypt. The research protocol was approved by the institutional review board (IRB) at the University of Louisville and Mansoura university as well as all methods were performed in accordance with the relevant guidelines and regulations and the patients informed consent was obtained. For the patients who passed away, an informed consent was obtained from legal guardian/Next of kin for the dead patients. These databases include 200 subjects tested as COVID-19 positive, 100 from patients who eventually died from the infection and 100 patients who ultimately recovered. These databases comprise a heterogeneous collection of digital X-ray images, which was the primary motivation to develop rotation, scale, and translation invariant MGRF model from which we extract the proposed imaging markers to grade the severity of lung infection in COVID-19 patients. We did our experiments using the merged datasets from all three databases to overcome the issue of data balance as the number of subjects for the two classes, (high and low severity cases), in each database was different. The dead cases had been confirmed based on the following radiology protocol^33^: (i) Bilateral and predominantly peripheral opacifications and/or consolidations were rated as typical for a COVID-19 infection. (ii) A distribution pattern with opacifications and/or consolidations limited to one pulmonary lobe consistent with lobar pneumonia was rated as non-typical for a COVID-19 infection. All changes that could not be classified as non-typical or typical were rated as indeterminate. In a subgroup of these patients with indeterminate findings, soft criteria for a possible COVID-19 infection were defined as the unilateral presence of predominantly peripheral.

### Proposed computer aided diagnostic (CAD) system

**Figure 1:**
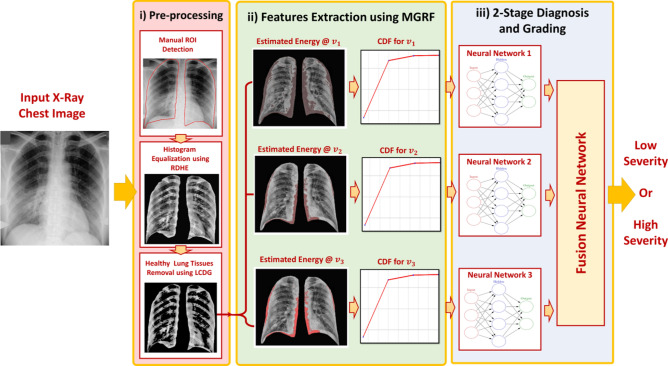
The pipeline of the proposed CAD system for COVID-19 diagnosis and grading.

The proposed CAD system to detect the severity of lung infection is shown in Fig. 1. The CAD system consists of three major steps: (i) preprocessing steps to improve contrast of the X-ray images and identify the region of interest in order to enhance diagnostic accuracy of subsequent steps; (ii) modeling the appearance of infected chest tissue using a new Markov–Gibbs random field (MGRF) constructed to be invariant under rotation, translation, and change of scale; and (iii) a neural network (NN)-based fusion and diagnostic system to determine whether the grade of lung infection is low or high.

#### Data preprocessing

**Figure 2:**
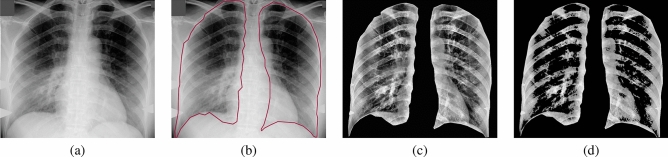
Illustration steps for the first two preprocessing steps in the proposed CAD system: (**a**) original X-ray image, (**b**) roughly segmented lung region, (**c**) enhanced contrast of lung region, and (**d**) extracted candidate of abnormal tissues.

To improve the accuracy of the proposed approach, we manually segmented the lung region from the original X-ray image, Fig. 2a, as demonstrated in Fig. 2b. The second step is to enhance lung tissue contrast, for which we use regional dynamic histogram equalization (RDHE)^34^. Proper analysis of the type of noise present in the chest X-ray image may help to select proper denoising methods, which preserve the important texture information while reducing the noise^35,36^. The RDHE approach divides the image into blocks *x* rows high by *y* columns wide. Then, dynamic histogram equalization is applied within each block to adaptively enhance the contrast. Therefore, the image histogram is remapped by block, and pixel values are adjusted relative to the other pixels in their $x\times y$ neighborhood. The contrast-enhanced X-ray image resulting from the RDHE approach is illustrated in Fig. 2c.

The third preprocessing step is to identify and mask off the healthy lung tissues from the infected tissues. This step narrows the search space to focus only on the abnormal tissues and serves to increase the diagnostic accuracy of the CAD system. To achieve this step, we use our previously published methodology^37^ that considers both the spatial interaction between nearby image pixels and the intensity distribution of those pixels within the lung region of interest. We follow the conventional description of the MGRF model in terms of independent signals (images) and interdependent region labels (segmentations); yet we focus on more accurate model identification^37^. Each image segment corresponds to a single dominant mode of the empirical distribution (i.e. histogram) of gray levels. To identify the dominant modes, each image histogram is considered to be sampled from a linear combination of discrete Gaussians (LCDG) distribution^37^. We fit an initial LCDG model to the empirical distribution using a modified expectation-maximization (EM) algorithm^37^. Free parameters of the LCDG to be optimized are the number of discrete Gaussian components and their respective weights (positive and negative), shape, and scale parameters. Then, the initial LCDG-based segmentation is iteratively refined using the MGRF model with its analytically estimated potentials^37^. Figure 2d shows the extracted pathological tissues using our proposed algorithm. Additional details can be found in El-Baz et al.^37^.

#### Rotation, scale, and translation invariant MGRF model

**Figure 3:**
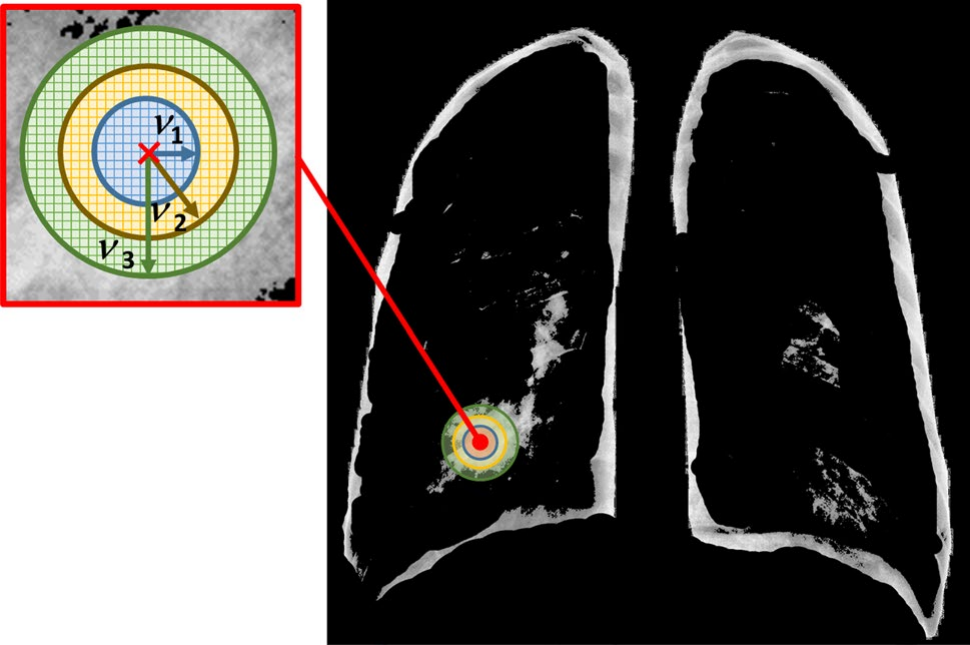
Illustration of rotation and translation invariant central-symmetric neighborhood sets for the three different radii $(\nu - 0.5, \nu + 0.5]$; $\nu = 1, \ldots , 3$.

We constructed the proposed MGRF model in order that the image need not be aligned with any particular frame of reference in order to use it to grade the severity of lung infection (low vs. high). To construct the appearance of the infected lung regions, we consider the X-ray images as samples from a piecewise stationary MGRF with a central-symmetric system of pixel-pixel interactions. Let $\mathbf {n}_{\nu }$ denote a set of central-symmetric pixel neighborhoods indexed by $\nu \in \{1, \ldots , N\}$. Each $\mathbf {n}_{\nu }$ is a set of coordinate offsets $(\xi , \eta )$ specified by a semi-open interval of interpixel distances $(d_{\nu ,\min }, d_{\nu ,\max }]$ such that the $\mathbf {n}_{\nu }$-neighborhood of pixel (*x*, *y*) comprises all pixels $(x^\prime , y^\prime )$ such that $d_{\nu ,\min } < \sqrt{(x - x^\prime )^2 + (y - y^\prime )^2} \le d_{\nu ,\max }$. A neighborhood system corresponding to $d_{\nu ,\min } = \nu - {1\over 2}$ and $d_{\nu ,\max } = \nu + {1\over 2}$, $\nu \in \{1, 2, 3\}$, is illustrated in Fig. 3. Associated with the neighborhood system is a set of $N + 1$ Gibbs potential functions of gray value and gray value co-occurrences $V_0:Q \rightarrow \mathbb {R}$ and $V_\nu :Q\times Q \rightarrow \mathbb {R}$, $\nu \in \{1, \ldots , N\}$, where *Q* is the range of pixel gray levels, e.g. $Q = \{0, \ldots , 255\}$ in the case of 8-bit images.

For a given image/label map pair $(\mathbf {g}_t, \mathbf {m}_t)$ from our training set *S*, $t \in \{1, \ldots , T\}$, let $\mathbf {R}_t = \{(x,y) \mid m_t(x,y) = \mathsf {ob}\}$ denote the subset of the pixel lattice supporting the infected lung region. Denote the set of $\mathbf {n}_\nu$-neighboring pixels restricted to $\mathbf {R}_t$ by

$$\begin{aligned} \mathbf {C}_{\nu ,t} = \left\{ (x, y, x^\prime , y^\prime ) \, \big |\, (x,y)\in \mathbf {R}_t \wedge (x^\prime ,y^\prime ) \in \mathbf {R}_t \wedge (x-x^\prime , y-y^\prime ) \in \mathbf {n}_\nu \right\} . \end{aligned}$$

Finally let $f_{0,t}$ and $f_{\nu ,t}$, $\nu \in \{1, \ldots , N\}$ denote empirical probability distributions (i.e., relative frequency histograms) of gray values and gray value co-occurrences in the training infected region from the X-ray image $\mathbf {g}_t$,

1 $$\begin{aligned}&f_{0,t}(q) = \vert \mathbf {R}_t\vert ^{-1} \left| \{(x,y)\in \mathbf {R}_t \mid \mathbf {g}_t(x,y) = q\} \right| ; \end{aligned}$$

2 $$\begin{aligned}&f_{\nu ,t}(q,q^\prime ) = \vert \mathbf {C}_{\nu ,t}\vert ^{-1} \left| \{(x,y,x^\prime ,y^\prime ) \in \mathbf {C}_{\nu ,t} \mid \mathbf {g}_t(x,y) = q \wedge \mathbf {g}_t(x^\prime ,y^\prime ) = q^\prime \} \right| . \end{aligned}$$

The joint probability of object pixels in image $\mathbf {g}_t$ according to the MGRF model is given by the Gibbs distribution

3 $$\begin{aligned} \begin{array}{lll} P_t &{} = &{} Z_{t}^{-1} \exp \left( \sum \limits _{(x,y)\in \mathbf {R}_t} \left( V_0\left( \mathbf {g}_t(x,y)\right) + \sum \limits _{\nu =1}^{N} \sum \limits _{(\xi ,\eta )\in \mathbf {n}_{\nu }} V_{\nu }\left( \mathbf {g}_t(x,y),\mathbf {g}_t(x+\xi ,y+\eta )\right) \right) \right) \\ &{} = &{} Z_{t}^{-1} \exp \left( \vert \mathbf {R}_t\vert \left( \mathbf {V}_{0,t}^\mathsf {T}\mathbf {F}_{0,t} + \sum \limits _{\nu =1}^{N}\rho _{\nu ,t}\mathbf {V}_{\nu ,t}^\mathsf {T} \mathbf {F}_{\nu ,t} \right) \right) , \end{array} \end{aligned}$$

where $\rho _{\nu ,t}= \vert \mathbf {C}_{\nu ,t}\vert / \vert \mathbf {R}_t\vert$ is an average cardinality of $\mathbf {n}_{\nu }$ over the sublattice $\mathbf {R}_t$.

Assuming lungs having the same pathology exhibit similar morphology in X-ray images, then we may approximate the previous expressions by their averages over the training set *S*: $|\mathbf {R}_t| \approx R_\mathsf {ob}$ and $|\mathbf {C}_{\nu ,t}| \approx C_{\nu ,\mathsf {ob}}$. Here $R_\mathsf {ob} = {1\over T}\sum _{t=1}^{T}|\mathbf {R}_t|$ and $C_{\nu ,\mathsf {ob}} = {1\over T}\sum _{t=1}^{T}|\mathbf {C}_{\nu ,t}|$. If we assume further that the observations in *S* are statistically independent (e.g., each X-ray is taken from a different patient), the expression for joint probability of object pixels may be likewise simplified^38^:

$$\begin{aligned} P_\mathbf {S}=\frac{1}{Z} \exp \left( TR_\mathsf {ob} \left( \mathbf {V}_{0}^\mathsf {T} \mathbf {F}_{0}+ \sum \limits _{\nu =1}^{N}\rho _{\nu }\mathbf {V}_{\nu }^\mathsf {T} \mathbf {F}_{\nu } \right) \right) . \end{aligned}$$

Here, $\rho _\nu = C_{\nu ,\mathsf {ob}} / R_\mathsf {ob}$, and the probability vectors $\mathbf {F}_{\mathrm {pix},\mathsf {ob}}$ and $\mathbf {F}_{\nu ,\mathsf {ob}}$ are the averages of the relative frequency histograms and normalized gray level co-occurrence matrices, respectively, over all objects in the training set. The problem of zero empirical probabilities, which can arise when a relatively small volume of the training data is available to identify the MGRF model, is dealt with using pseudocounts. Then Eqs. 1 and 2 are modified as follows

4 $$\begin{aligned}&f_{0,t}(q) = \frac{\left| \{(x,y)\in \mathbf {R}_t \mid \mathbf {g}_t(x,y) = q\} \right| + \varepsilon }{\vert \mathbf {R}_t\vert + Q\varepsilon } \end{aligned}$$

5 $$\begin{aligned}&f_{\nu ,t}(q,q^\prime ) = \frac{\left| \{(x,y,x^\prime ,y^\prime ) \in \mathbf {C}_{\nu ,t} \mid \mathbf {g}_t(x,y) = q \wedge \mathbf {g}_t(x^\prime ,y^\prime ) = q^\prime \} \right| + \varepsilon }{\vert \mathbf {C}_{\nu ,t}\vert + Q^2\varepsilon }. \end{aligned}$$

The Bayesian quadratic loss estimate suggests using the offset $\varepsilon = 1$, while a more conservative approach^38^ suggests using $\varepsilon = 1/Q$ in Eq. 4 and $\varepsilon = 1/Q^2$ in Eq. 5.

Using the same analytical approach as in Ref.^38^, the Gibbs potential functions are approximated using the centered, training-set average, normalized histograms and co-occurrence matrices:

6 $$\begin{aligned} \begin{array}{llll} V_{0}(q) &{} = &{} \left( f_{0}(q)-\frac{1}{Q} \right) ; \\ V_{\nu }(q,q^{\prime }) &{} = &{} \left( f_{\nu }(q,q^{\prime })-\frac{1}{Q^{2}} \right) . \end{array} \end{aligned}$$

Using the above estimated potentials, we can calculate the Gibbs energy of the infected lung region $\mathbf {b}$ in an X-ray image $\mathbf {g}$ as follows:

7 $$\begin{aligned} E(\mathbf {g},\mathbf {b}) = \mathbf {V}_{0}^\mathsf {T} \mathbf {F}_{0}(\mathbf {g}, \mathbf {b}) + \sum _{\nu \in \mathbf {N}^\prime }\mathbf {V}_{\nu }^\mathsf {T} \mathbf {F}_{\nu }(\mathbf {g}, \mathbf {b}). \end{aligned}$$

Here, $\mathbf {N}^\prime$ is a selected top-rank index subset of the neighborhoods, and the empirical probability distributions $F_0$ and $F_{\nu }$ are calculated over the object pixels $\mathbf {b}$ of $\mathbf {g}$.

To summarize, the whole training approach is as follows:

1. Read all infected regions from the training data having class “severe” lung infection.
2. Calculate the co-occurrence of the image signal at various radii ($\nu$1, $\nu$2, and $\nu$3).
3. Normalize the co-occurrence frequency ($f_{\mathrm {pix},\mathsf {ob}}(q)$).
4. Estimate the Gibbs potential ($V_{\mathrm {pix},\mathsf {ob}}(q)$) by using Eq. 6.
5. Use Eq. 7 to calculate the Gibbs Energy ($E(\mathbf {g},\mathbf {b})$) for the training subjects.

## NN-based fusion and diagnostic system

**Figure 4:**
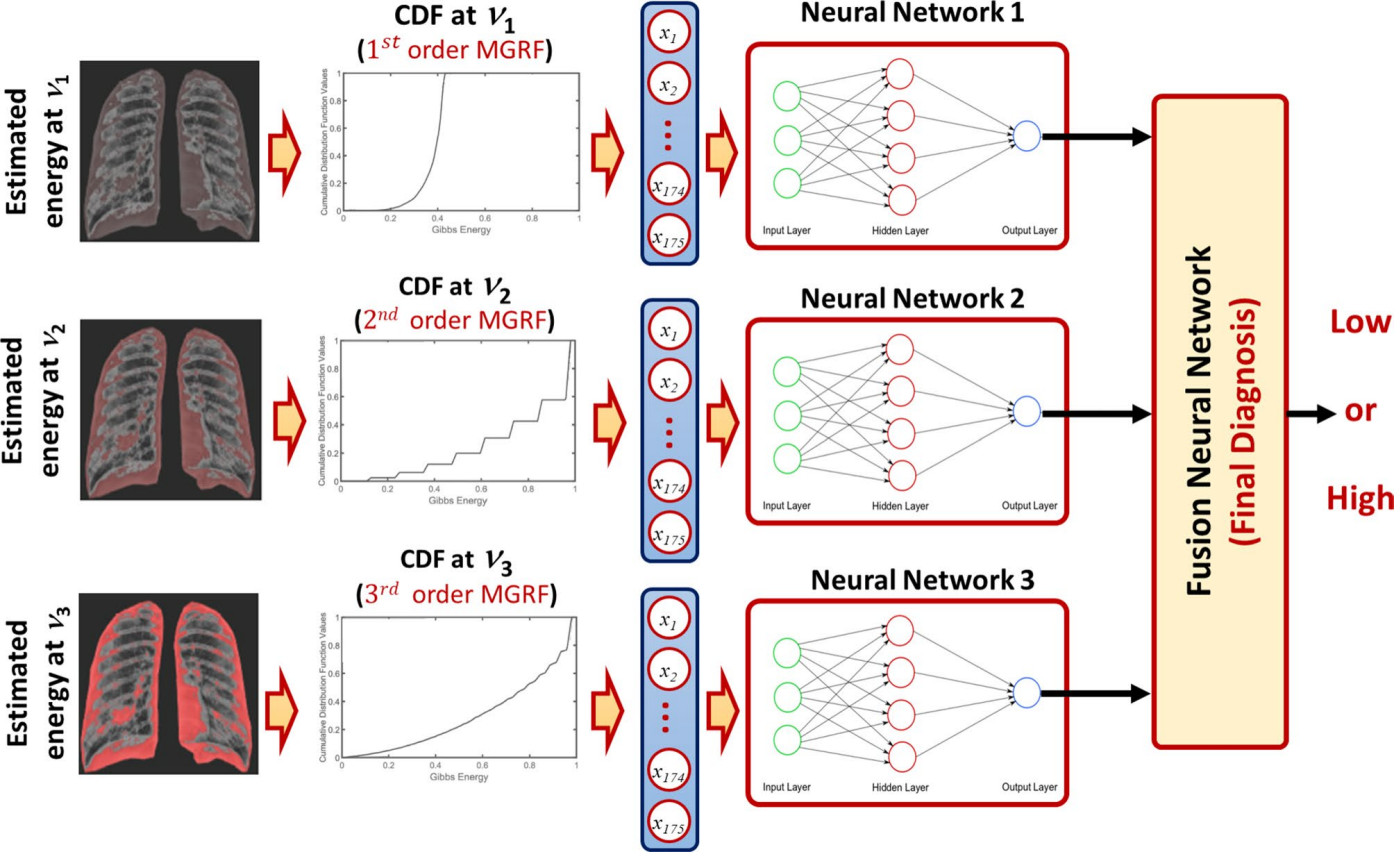
The proposed NN-based fusion diagnostic system.

A new NN system that can fuse the diagnostic results from the three estimated Gibbs energy at three different radii is developed. The proposed NN-based model consists of four blocks as illustrated in Fig. 4. Three of them are fed with the three different cumulative distribution functions (CDFs) of the estimated Gibbs energy, then the results of the three blocks are fused into the last block to decide the final decision of the input X-ray image. We use a backpropagation approach to train the proposed NN-based diagnostic system as follows:

1. Randomly initialize the weights of the proposed NN-network.
2. Compute the output of each neuron in the hidden and output layers.
3. Update the weights of the proposed NN-network using the batch-mode backpropagation approach.
4. Repeat steps 2 and 3 until there are no significant changes in the NN-network weights.

In order to tune the hyper-parameters used in our proposed NN system, a hyper-parameters estimation approach is used. The parameters to be estimated are the number of bins used to calculate CDF, number of hidden layers in the NN model, number of neurons in the hidden layer, and finally the activation function used to calculate the output of each neuron. We ran several experiments using random values for these parameters to estimate their optimal values using training data. All the results that are demonstrated in the “Experimental results” section have been obtained using the following setting: to handle all energy values, the chosen value for the number of CDF bins is 175; the number of hidden layers in NN 1, NN 2, and fusion NN is one, while for NN 3, there are no hidden layers (searching from 0 to 10); the number of neurons per hidden layer is 50, 20, and 2 for NN 1, NN 2, and Fusion NN, respectively (searching from 1 to 200); and finally, the sigmoid activation function has been selected after also considering the tangent and softmax activation functions.

## Experimental results

**Figure 5:**
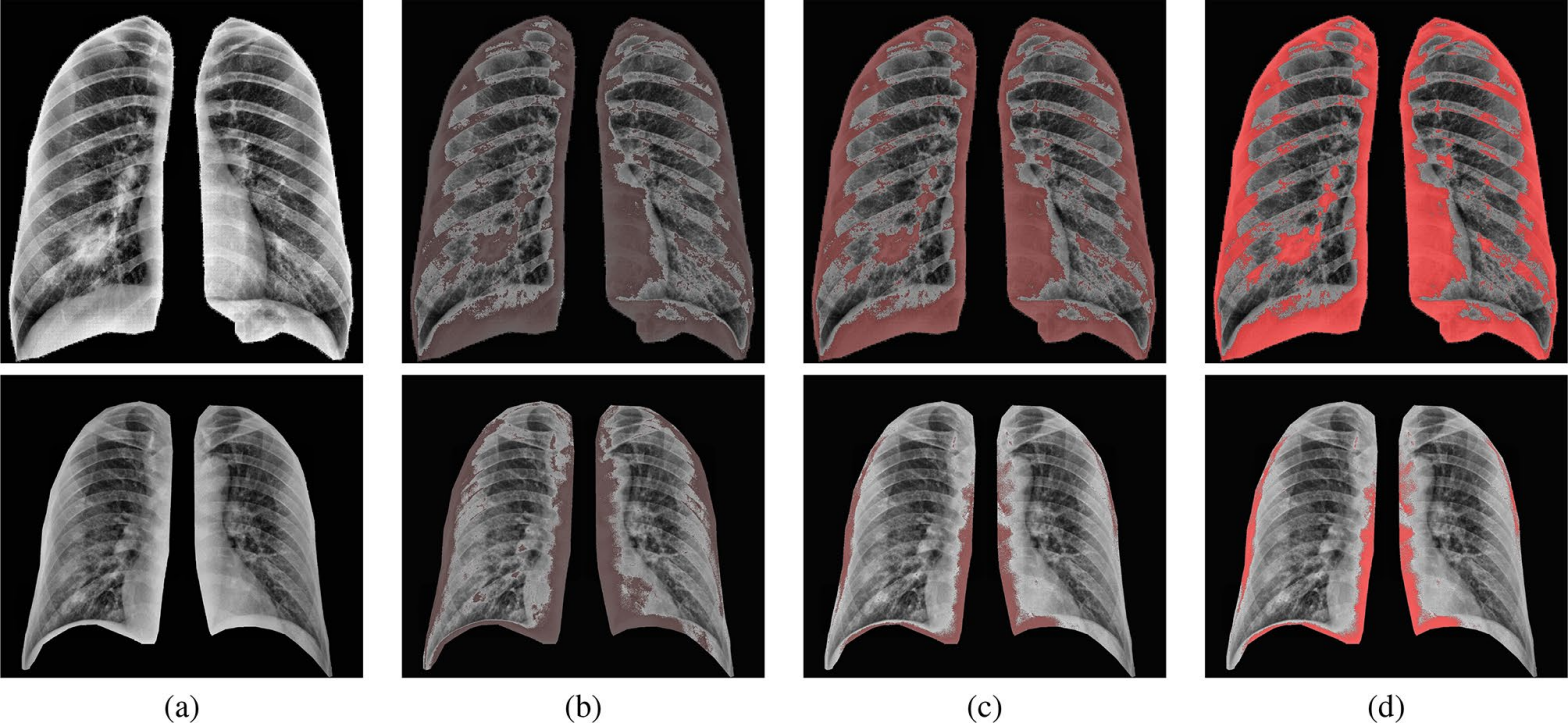
Illustration of the estimated Gibbs energy for two cases: high severity case (upper raw) and low severity case (lower raw). (**a**) equalized X-ray image, (**b**) estimated energy at $\nu$1, (**c**) estimated energy at $\nu$2, and (**d**) estimated energy at $\nu$3.

**Figure 6:**
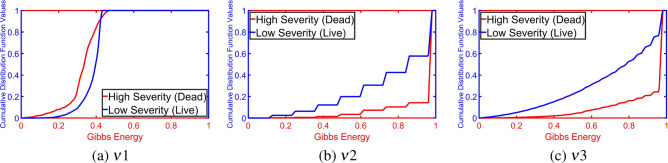
Estimated CDFs at three different radii for two different subjects at two different severity levels.

**Figure 7:**
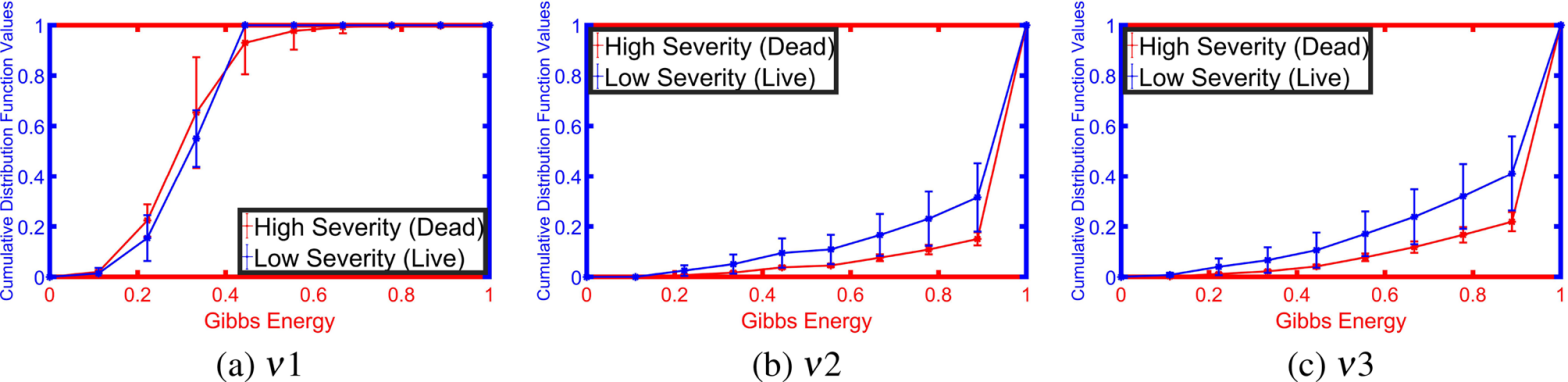
Estimated average CDFs for low and high lung infection severity at three different radii.

**Table 1:** Diagnostic accuracy of the proposed CAD system. Feature selection had a significant impact on classifier performance with Friedman test $\chi ^2 = 32.7$, 3 d.f., $p = 3\times 10^{-7}$. *V*: Wilcoxon signed rank statistic of performance compared to complete system; *p*: Associated Bonferroni-corrected *p*-value.

	Sensitivity	Specificity	DSC	Accuracy	*V*	*p*
**Performance of the proposed whole CAD system**						
LOSO	100% ± 0.00	100% ± 0.00	100% ± 0.00	100% ± 0.00	–	–
Tenfold	100% ± 0.00	99% ± 1.00	99.50% ± 0.50	99.50% ± 0.50		
Fourfold	100% ± 0.00	98% ± 2.00	99% ± 1.00	99% ± 1.00		
Twofold	100% ± 0.00	97% ± 3.00	98% ± 2.00	98% ± 2.00		
**Performance of the proposed CAD system when using only the estimated energy at** $\nu$**1**						
LOSO	76% ±4.29	96% ± 1.97	75% ± 4.27	86% ± 2.37	118	0.0033
Tenfold	74% ± 1.26	98% ± 4.21	83.61% ± 9.15	86% ± 7.37		
Fourfold	71% ± 8.28	98% ± 2.31	81.87% ± 5.05	84.50% ± 3.41		
Twofold	71% ±4.24	99% ± 1.41	80.87% ± 2.14	80.30% ± 1.41		
**Performance of the proposed CAD system when using only the estimated energy at** $\nu$**2**						
LOSO	81% ± 3.94	94% ± 2.39	79.33% ± 3.93	87.5% ± 2.28	136	0.0014
Tenfold	79% ± 1.29	94% ± 5.07	85.45% ± 6.23	87% ± 4.83		
Fourfold	80% ± 8.86	93% ± 6.31	83.83% ± 6.15	86.50% ± 3.41		
Twofold	77% ± 1.41	91% ± 2.82	82.16% ± 2.20	85.50% ± 2.12		
**Performance of the proposed CAD system when using only the estimated energy at** $\nu$**3**						
LOSO	97% ±1.71	94% ± 2.39	95% ± 1.85	95.5% ± 1.43	136	0.0014
Tenfold	93% ± 8.23	97% ± 4.83	94.78% ± 5.56	95% ± 5.27		
Fourfold	92% ± 1.13	95% ± 3.83	93.16% ± 7.82	93.50% ± 7.19		
Twofold	91% ±7.07	95% ± 1.41	92.79% ± 3.20	93% ± 2.82		

To highlight the innovation in our approach, we demonstrate the Gibbs energy calculated at three radii as a color map fused over the X-ray images. One example for which it holds, it is clear from Fig. 5 that the Gibbs energy in cases of high severity of COVID-19 pneumonia is high compared with the Gibbs energy for low-severity COVID-19 pneumonia. Since the collected X-ray images have different resolutions, we use CDF as a new scale-invariant representation to the estimated Gibbs energy which makes it suitable for all data collection protocols as shown in Fig. 6. To highlight the advantage of the proposed Gibbs energy as a new discriminatory image marker, we calculate the average CDFs with a demonstration of the standard deviation at each point for both classes (high severity vs. low severity). As is clear from Fig. 7, the CDFs are rather distinctive which allows for straightforward classification by the proposed NN-based classifier. The output of the CAD system was an assessment of the severity of pneumonia in COVID-19 patients with two levels: a low severity of infection (“low”) or high severity of infection (“high”) as shown in Fig. 4. This was compared to the ground truth of the 200 clinical cases collected, 100 of which were from patients who died of COVID-19 and 100 of which recovered. Accurate system outputs include an assessment of “low” in a case that recovered and an output of “high” in a case that died. To confirm the accuracy of the proposed NN classification and fusion system, leave-one-subject-out (LOSO), tenfold, fourfold, and a twofold cross-validation approaches are performed on our datasets as demonstrated in Table 1. We use the following objective metrics to measure the accuracy of the proposed NN-based fusion system: (i) sensitivity, (ii) specificity, (iii) accuracy, and (iv) Dice similarity coefficient (DSC). As demonstrated in Table 1, the proposed system has achieved $100\%$ accuracy with the LOSO validation test and $98.00\% \pm 2.00\%$ for a twofold validation test (real-life scenario), all of which confirm the high accuracy of the proposed CAD system.

To highlight the contribution of each Gibbs energy at each radius, we construct an NN-based classifier using the estimated Gibbs energy at each radius. As is clear from Table 1, the NN-classifier based on the estimated Gibbs energy at $\nu$3 demonstrates the highest accuracy compared with the classification accuracies based on the estimated Gibbs energy at $\nu$2 and $\nu$1. Also, it is worth mentioning that fusing the three estimated Gibbs energies by using the NN-Based classification system achieves higher accuracy compared with classification accuracies based on each single estimated Gibbs energy. Finally, to highlight the accuracy of the proposed NN-based fusion system, we compare its accuracy with support vector machine (SVM), random forest, naive Bayes, K-nearest neighbors (KNN), and decision trees classifiers. Table 2 clearly shows that the NN-based classification and fusion system has achieved the highest accuracy compared with other approaches.

**Table 2:** Diagnostic accuracy using different classification systems. Feature selection had a significant impact on classifier performance with Friedman test $\chi ^2 = 35.3$, 5 d.f., $p = 1.3\times 10^{-6}$. *V*: Wilcoxon signed rank statistic of performance compared to complete system; *p*: Associated Bonferroni-corrected *p*-value.

	Sensitivity	Specificity	DSC	Accuracy	*V*	*p*
**SVM-based CAD system**						
LOSO	86% ±3.48	94% ± 2.39	84% ± 3.49	90% ± 2.01	136	0.0024
Tenfold	78% ± 9.19	97% ± 4.83	85.96% ± 6.06	87.50% ± 4.86		
Fourfold	85% ± 1.41	92% ± 5.65	88.11% ± 1.76	88.50% ± 2.20		
Twofold	83% ±3.82	91% ± 3.83	86.44% ± 1.29	87% ± 1.15		
**Random forest-based CAD system**						
LOSO	76% ±4.29	96% ± 1.97	75% ± 4.27	86% ± 2.37	118	0.0054
Tenfold	74% ± 1.26	98% ± 4.21	83.61% ± 9.15	86% ± 7.37		
Fourfold	71% ± 8.28	98% ± 2.31	81.87% ± 5.05	84.50% ± 3.41		
Twofold	71% ±4.24	99% ± 1.41	80.87% ± 2.14	80.30% ± 1.41		
**Naive Bayes-based CAD system**						
LOSO	84% ±3.68	94% ± 2.38	82.33% ± 3.68	89% ± 2.19	136	0.0024
Tenfold	80% ± 1.05	97% ± 4.83	87.13% ± 7.10	88.50% ± 5.79		
Fourfold	77% ± 6.00	97% ± 2.00	85.46% ± 4.36	87% ± 3.46		
Twofold	77% ±4.24	95% ± 1.41	84.58% ± 2.03	86% ± 1.41		
**KNN-based CAD system**						
LOSO	80% ±4.02	99% ± 1.00	79.66% ± 4.01	89.50% ± 2.04	127.5	0.0114
Tenfold	75% ± 8.87	100% ± 0.00	85.49% ± 5.88	87.50% ± 4.43		
Fourfold	71% ± 1.10	100% ± 0.00	82.61% ± 7.43	85.50% ± 5.50		
Twofold	70% ±0.00	100% ± 0.00	82.35% ± 0.00	85% ± 0.00		
**Decision Trees-based CAD system**						
LOSO	80% ±4.02	99% ± 1.00	79.66% ± 4.01	89.50% ± 2.04	127.5	0.0114
Tenfold	75% ± 8.87	100% ± 0.00	85.49% ± 5.88	87.50% ± 4.43		
Fourfold	71% ± 1.10	100% ± 0.00	82.61% ± 7.43	85.50% ± 5.50		
Twofold	70% ±0.00	100% ± 0.00	82.35% ± 0.00	85% ± 0.00		

Statistical significance of the choice of feature set or classifier architecture on diagnostic performance was assessed using Friedman rank sum tests^39–42^. Post hoc comparison of our proposed NN-based classifier with the alternatives was done using Wilcoxon signed rank tests with Bonferroni correction to the estimated $p-$values. Feature selection had a significant impact on classifier performance (Table 1), with Friedman test $\chi ^2 = 32.7$, 3 d.f., $p = 3\times 10^{-7}$. The fused feature set was shown to be preferable to v1 (Wilcoxon $V = 118$, Bonferroni corrected $p = 0.0033$), v2 ($V = 136$, $p = 0.0014$), and v3 ($V = 136$, $p = 0.0014$). Different classifier architectures also produced significantly different results (Table 2), with $\chi ^2 = 35.3$, 5 d.f., $p = 1.3\times 10^{-6}$. The proposed NN-based classifier outperformed SVM ($V = 136$, $p = 0.0024$), random forest ($V = 118$, $p = 0.0054$), naive Bayes classsifier ($V = 136$, $p = 0.0024$), KNN ($V = 127.5$, $p = 0.0114$), and the decision tree ($V = 127.5$, $p = 0.0114$).

## Discussion and conclusion

ARDS is the most common and severe pulmonary complication in COVID-19 patients. It is an acute hypoxemic respiratory failure that requires oxygen and ventilation therapy including intubation and invasive ventilation. Clinically patients may have dyspnea, tachypnea (respiratory rate $\ge$ 30 breaths per minute), decreased peripheral oxygen saturation $\text {SpO}_2 \le 93\%$, poor oxygenation with the ratio of the partial pressure of arterial oxygen to fraction of inspired oxygen $\text {PaO}_2 / \text {FiO}_2 < 300$ mmHg, or lung infiltrates greater than 50% within 48 h^9^. ARDS occurred in 20% of hospitalized patients and 61% of ICU patients in Zhongnan Hospital in Wuhan^3,4^. ARDS occurs when capillaries in the lung leak fluid into the alveoli, thereby impairing gas exchange in the lung and reducing oxygen uptake into the systemic arterial circulation. The consequent decrease in blood oxygen levels can be directly life-threatening, leading to multi-organ failure. Respiratory support of COVID-19 may use invasive or non-invasive methods to force oxygen into the airways under pressure. Invasive ventilation uses an endotracheal tube to feed oxygen directly into the lungs. Non-invasive methods employ such devices as continuous positive airway pressure (CPAP) and oxygen hoods; there is no use of an internal tube, and they are used in the management of less severe cases.

Despite being vital for supporting respiration in patients with ARDS, ventilators are in short supply in hospitals. According to Imperial College London, 30% of patients diagnosed with COVID-19 are strongly recommended to be admitted to the hospitals, with a significant fraction of those patients also requiring respiratory support. As the pandemic spreads across the world, many countries stopped exporting ventilators^43,44^. The paucity of ventilators is even more acute in under developed and developing countries in South America, Asia, and Africa.

High-pressure ventilation may cause lung injury, also called barotrauma or ventilator-induced lung injury (VILI). Even non-invasive ventilation carries some risk, as stress and strain associated with high tidal volumes may cause patient self-induced lung injury (P-SILI). The additional inflammation due to VILI or P-SILI may lead to aggravation of pulmonary edema and worsening of the very respiratory distress that ventilation was intended to treat. There is also the risk of heart failure, hypervolemia, and multi organ dysfunction, alone or in combination^45^. Unfortunately, COVID-19 patients who are admitted to the ICU and require mechanical ventilation show strikingly high rates of mortality, ranging from 50 to 97% early in the pandemic^46–51^. A more recent study from Emory University showed lower but still dramatic mortality rates of 36% in ICU patients requiring mechanical ventilation and 30% in all COVID-19 patients admitted to the ICU^52^.

Accurate and rapid diagnosis of COVID-19 pneumonia severity is very challenging for radiologists as the disease has rapidly spread across the globe. Based on the results demonstrated in this study, AI systems, especially those based on deep learning, are promising tools to assist initial screening by radiologists. It could decrease workload, improve diagnostic accuracy, and enable appropriate treatments and ventilation management of COVID-19 patients. In the case of a pandemic as we now face, medical resources are seriously strained and must be used as efficiently as possible. Rapid diagnosis and accurate prognosis are essential. The AI-based method shows great potential to quantify disease severity and could be used to inform treatment decision-making in patients with COVID-19. AI in concert with thoracic imaging and other clinical information (epidemiology, PCR, clinical symptoms, and laboratory indicators) can effectively improve clinical outcomes^53^. AI can increase the utility of chest X-ray imaging beyond first-line diagnostic imaging and into the areas of risk stratification, monitoring of clinical course, and selection between management approaches, such as invasive vs. non-invasive ventilation, for COVID-19 patients. Multimodal data, be they clinical, epidemiological, or potentially molecular data, can by fused with imaging data in an AI framework to build systems to detect and treat COVID-19 patients and potentially to contain its spread^54^. Moreover, we plan to work on X-ray scans/data that are collected at different time points to evaluate the progressing of the infection/pneumonia with the treatment course.

In conclusion, the results herein demonstrate the feasibility of using AI with chest X-ray imaging data to determine the severity of lung involvement in cases of COVID-19. Severity of pneumonia on chest X-ray correlated highly with mortality in this study, and thus this CAD system can potentially also be used to predict mortality in COVD-19 patients.

## Data Availability

Materials, data, and associated protocols will be available to readers after the manuscript being accepted.
